# Cross-Protective Potential and Protection-Relevant Immune Mechanisms of Whole Inactivated Influenza Virus Vaccines Are Determined by Adjuvants and Route of Immunization

**DOI:** 10.3389/fimmu.2019.00646

**Published:** 2019-03-29

**Authors:** Yoshita Bhide, Wei Dong, Inta Gribonika, Daniëlle Voshart, Tjarko Meijerhof, Jacqueline de Vries-Idema, Stephen Norley, Kate Guilfoyle, Sarah Skeldon, Othmar G. Engelhardt, Louis Boon, Dennis Christensen, Nils Lycke, Anke Huckriede

**Affiliations:** ^1^Department of Medical Microbiology and Infection Prevention, University Medical Center Groningen, University of Groningen, Groningen, Netherlands; ^2^Department of Microbiology and Immunology, Institute of Biomedicine, Gothenburg University, Gothenburg, Sweden; ^3^Department of Infectious Diseases, Robert Koch Institute, Berlin, Germany; ^4^Division of Virology, National Institute for Biological Standards and Control (NIBSC), Medicines and Healthcare products Regulatory Agency (MHRA), Potters Bar, United Kingdom; ^5^Bioceros, Utrecht, Netherlands; ^6^Adjuvant Research, Department of Infectious Diseases Immunology, Statens Serum Institut (SSI), Copenhagen, Denmark

**Keywords:** whole inactivated virus (WIV) influenza vaccines, liposome-based adjuvants, protein-based adjuvants, cross protection, non-neutralizing serum antibodies, CD4 T cells

## Abstract

Adjuvanted whole inactivated virus (WIV) influenza vaccines show promise as broadly protective influenza vaccine candidates. Using WIV as basis we assessed the relative efficacy of different adjuvants by carrying out a head-to-head comparison of the liposome-based adjuvants CAF01 and CAF09 and the protein-based adjuvants CTA1-DD and CTA1-3M2e-DD and evaluated whether one or more of the adjuvants could induce broadly protective immunity. Mice were immunized with WIV prepared from A/Puerto Rico/8/34 (H1N1) virus intramuscularly with or without CAF01 or intranasally with or without CAF09, CTA1-DD, or CTA1-3M2e-DD, followed by challenge with homologous, heterologous or heterosubtypic virus. In general, intranasal immunizations were significantly more effective than intramuscular immunizations in inducing virus-specific serum-IgG, mucosal-IgA, and splenic IFNγ-producing CD4 T cells. Intranasal immunizations with adjuvanted vaccines afforded strong cross-protection with milder clinical symptoms and better control of virus load in lungs. Mechanistic studies indicated that non-neutralizing IgG antibodies and CD4 T cells were responsible for the improved cross-protection while IgA antibodies were dispensable. The role of CD4 T cells was particularly pronounced for CTA1-3M2e-DD adjuvanted vaccine as evidenced by CD4 T cell-dependent reduction of lung virus titers and clinical symptoms. Thus, intranasally administered WIV in combination with effective mucosal adjuvants appears to be a promising broadly protective influenza vaccine candidate.

## Introduction

Vaccination is the cornerstone for the prevention of influenza (1). Current influenza vaccines predominantly mediate strain specific protection by eliciting neutralizing antibody responses to the globular head region of hemagglutinin (HA), one of the surface glycoproteins of the virus. They do not provide protective immunity against strains not included in the vaccine (1, 2). New virus strains regularly emerge through antigenic drift, the phenomenon responsible for recurrent epidemics. Moreover, zoonotic influenza virus subtypes pose a serious pandemic threat, as exemplified by pandemic H1N1(2009) and the potentially pandemic subtypes H5N1, H7N9, H10N8, or H5N6 (3–6). There is therefore an urgent need for broadly protective influenza vaccines which can prevent or at least mitigate infection by virus strains not included in the vaccine.

Whole inactivated virus (WIV) vaccines contain all the structural viral proteins and retain the conformation of native virus particles and as such make a promising basis for an influenza vaccine. Moreover, WIV has an intrinsic ability to activate innate immune responses, e.g., antigen presenting cells via Toll-like receptor 7 (TLR7) signaling (7). Although WIV was the first vaccine to be used, it was later replaced by split and subunit vaccines that were considered safer (8), despite WIV being superior at inducing immune responses in mice and naïve human beings (7, 9–12). Interest has recently refocused on WIV vaccines as studies have shown them capable of inducing a certain degree of cross-protection upon parenteral and mucosal vaccination (3, 13–16). However, a large amount of antigen was required to achieve protection and/or virus challenge was only performed shortly after immunization in these studies (16). One approach to reduce the dose of WIV needed would be to use adjuvants that might also improve the breadth of the immune responses (17–19).

There are various adjuvants under investigation for improving the immunogenicity of influenza vaccines (20). In this study, we compared the liposome-based adjuvants CAF01 and CAF09 and the protein-based adjuvants CTA1-DD and CTA1-3M2e-DD. These adjuvants were chosen because they were previously used successfully with several vaccine candidates, including influenza vaccines and are ready for or currently evaluated in clinical trials (21–38). The cationic adjuvant formulations, CAF01 and CAF09, are liposomes consisting of N,N′-dimethyl-N,N′-dioctadecylammonium (DDA) as delivery vehicle. For CAF01, α,α′-trehalose 6,6′-dibeheneate (TDB) acts as an immunomodulator and liposome-stabilizer, while CAF09 is stabilized and adjuvanted with monomycoloyl glycerol (MMG)-1 and contains the TLR3 ligand Poly(I:C) as an additional immunomodulator (21, 24). CAF01 and CAF09 have been shown to generate strong T cell and antibody responses, with particularly high IgG2a responses for CAF01 (21, 22, 37). CAF09 is furthermore capable of inducing potent CD8+ T cell responses against protein and peptide based antigens (24, 33, 37, 38). CAF01 can be administered parenterally while CAF09 is mainly administered intraperitoneally (i.p.,). However, there has been a number of studies which showed promising results when CAF09 was given mucosally (Christensen et al. unpublished data). Furthermore, CAF05, a predecessor adjuvant was successfully administered via mucosal route (39). This motivated us to administer CAF09 via intranasal route. CTA1-DD is a fusion protein consisting of the enzymatically active A1 subunit of cholera toxin and a dimer of an Ig binding element from *Staphylococcus aureus* protein A. It targets cells of the innate immune system which results in strongly enhanced humoral and cellular immune responses (27–29). Contrary to whole cholera toxin the mucosal CTA1-DD adjuvant is safe and non-toxic as found in non-human primates and it does not accumulate in the olfactory bulb and nerve following administration intranasally (i.n.) and, hence, cannot cause Bell's palsy (40). CTA1-3M2e-DD harbors an insert of three copies of the exterior domain of the M2 protein of influenza virus, M2e (26, 30).

We compared these adjuvants head-to-head to assess their relative potency in stimulating cross-reactive and cross-protective anti-influenza immunity in mice. In order to mimick the situation of antigenic drift and antigenic shift, mice were immunized intramuscularly (i.m.) or i.n. with A/Puerto Rico/8/34 (PR8) WIV with or without the different adjuvants and 2 weeks after the final immunization mice were challenged with homologous PR8, heterologous (H1N1)pdm09 or heterosubtypic X-31 (H3N2) virus to assess protection and several immune parameters. We observed that WIV administered i.n. with the mucosal adjuvants conferred much stronger cross-protection than parenterally administered WIV with or without adjuvant. Studies into the significance of different immune mechanisms for protection revealed that non-neutralizing serum antibodies and CD4 T cells were important for cross-protection while IgA, even when present in high levels, did not play a critical role. Thus, WIV administered i.n. in combination with effective mucosal adjuvants provided the strongest cross-protection against heterosubtypic influenza virus infections and appears to be a promising candidate for a broadly protective influenza vaccine.

## Materials and Methods

### Viruses and Vaccines

Live influenza viruses PR8 (H1N1), A/California/7/2009 (H1N1)pdm09, and X-31 (H3N2) (a reassortant strain carrying the HA of A/Aichi/2/68 and the internal proteins of PR8) were propagated in embryonated chicken eggs and were titrated on MDCK cells and in CB6F1 mice. Whole inactivated virus vaccines (WIV) were prepared from these viruses by inactivation with beta-propiolactone. Beta-propiolactone was removed in down-stream processing by concentration of the allantoic fluid and purification using sucrose gradient centrifugation. The WIV HA content (μg/ml) was determined by using Lowry protein assay and SDS-PAGE (colloidal blue staining) to establish total protein content and percentage HA, respectively, the HA content was then calculated. Quality and quantity of HA were confirmed by single radial immunodiffusion assay (41).

### Adjuvants

The liposomal adjuvants CAF01 and CAF09 were produced as described previously (42). The dose for both adjuvants was 300 μg per 50 μl for i.m. and 300 μg per 40 μl for i.n. administration. The protein adjuvants, CTA1-DD and CTA1-3M2e-DD, were produced by MIVAC Development AB, Sweden. The latter construct carried three copies of the extracellular domain of the influenza virus M2 protein (SLLTEVETPIRNEWGSRSNDSSD). Briefly, the fusion proteins were expressed in E. coli DH5 cells, transformed with the expression vector for the fusion protein, and grown in 500 ml cultures overnight in SYPPG medium with 100 ug/ml carbenicillin, at 37°C, as previously described (30). Endotoxin levels were below 100 EU/mg as verified by use of the Endosafe® testing system (Charles River). For both protein adjuvants, the concentration was 5 μg per 40 μl WIV. Filtered Dulbecco's phosphate buffered saline containing CaCl_2_ and MgCl_2_ (DPBS, GIBCO by Life Technologies^TM^) was used as a diluent.

### Animal Experiments

All animal experiments were approved by the Institutional Animal Care and Use Committee of the University of Groningen (IACUC-RUG, DEC 6923), or the Central Committee for Animal Experiments CCD of the Netherlands (AVD105002016599), the Animal Welfare and Ethics Review Body (AWERB) of the National Institute for Biological Standards and Controls (NIBSC), Potters Bar, UK (PPL 80/2537), or the IACUC of the University of Gothenburg, Sweden.

#### Adjuvant Comparison Study

Female CB6F1/OlaHsd (C57Bl/6 x BALB/c F1) mice aged 6–8 weeks were purchased from Envigo (The Netherlands). The mice were distributed randomly and housed in groups of six within individually ventilated cages (IVC) at the animal facility, receiving standard water and diet. Group sizes were determined using Piface software aiming at a power of 80%. Mice were vaccinated three times with 0.5 μg HA of PR8 (with or without the adjuvants) on days 0, 10, and 20 as described in Table 1. Mice from groups 1–3 received 50 μl PBS or vaccine i.m., 25 μl per hindlimb. Mice from groups 4–7 received respective vaccines i.n. in a volume of 40 μl, divided between the two nostrils. Vaccination and virus challenge were carried out under Isoflurane/O_2_ anesthesia. Three weeks after the 3rd vaccination (day 41), six mice from each group (of 18) were sacrificed to determine vaccine-induced immune responses. The remaining mice were challenged with 10^4.4^ TCID_50_/mouse of homologous PR8 virus, 10^3.3^ TCID_50_/mouse of heterologous (H1N1)pdm09 or 10^5.5^ TCID_50_/mouse of heterosubtypic X-31 (titers were chosen on the basis of titration experiments in CB6F1 mice). Six mice from each experimental group were sacrificed on day 3 post challenge to assess protection against virus replication in the lungs. The remaining six mice were observed until day 10 post challenge to assess clinical symptoms such as weight loss, ruffled fur and activity. The humane endpoint was set to a loss of >20% of the original weight from the day of challenge. Additionally, for the mechanistic experiments, a score sheet was used to follow the animals. Parameters such as weight loss, appearance (degree of ruffled fur, hunched back) and behavior of the animals (slow movements, difficulty in walking, circling, response to external stimulus) were recorded. These parameters were given scores from 1 to 4 for least to most severe. A cumulative score for a given day of 10 was considered to be the humane endpoint.

**Table 1:** Vaccination and challenge scheme.

**Groups**	**Vaccine (D 0, 10, 20)**	**Route**	**Vaccine dose (μg HA)**	**adjuvant dose (μg)**	**injection volume (μl)**	**Challenge D 41**
1	PBS	i.m.	–	–	50	
2	WIV	i.m.	0.5	–	50	
3	WIV+ CAF01	i.m.	0.5	300	50	
4	WIV	i.n.	0.5	–	40	
5	WIV+ CAF09	i.n.	0.5	300	40	A/PR/8/34
6	WIV+ CTA1-DD	i.n.	0.5	5	40	(H1N1)pdm09
7	WIV+ CTA1-3M2e-DD	i.n.	0.5	5	40	X-31

#### Adoptive Serum Transfer

Serum samples were collected from mice mock-immunized with PBS, or immunized with PR8 WIV i.n., WIV+ CAF09 or WIV+CTA1-3M2e-DD as described above. Two hundred microliter of pooled sera were administered i.p. to naïve mice. Mice were then challenged with 10^5.5^ TCID_50_/mouse of heterosubtypic X-31 virus 1 day post adoptive transfer. Mice from positive control group received serum samples from mice immunized with PR8 WIV and were challenged with PR8 live virus. Animals were followed for 14 days and clinical symptoms were assessed using the scoring system described above.

#### CD4 T Cell Depletion

Anti-CD4 antibody (200 μg/injection, clone GK 1.5 Bioceros, Utrecht, The Netherlands) was used for *in vivo* CD4 depletion, which was assessed by staining with FITC-labeled anti-CD4 (clone RM 4.4, Thermo Scientific). Female CB6F1 mice (aged 6–8 weeks) were immunized as described above (groups 1, 4, 5, 7 from Table 1) followed by a heterosubtypic challenge with X-31 virus (10^5.5^ TCID_50_/mouse). Mice were injected i.p. with the anti-CD4 antibody 1 day before, 1 and 7 days after challenge. Six animals/group were sacrificed on day 3 post challenge for assessment of lung virus titers while the remaining animals were followed for 14 days for clinical symptoms using the scoring system described above.

#### IgA Knockout Experiment

IgA knock-out mice (IgA KO; BALB/c background, males and females) were obtained from Margaret Conner, Baylor College of Medicine, Houston, TX, US and bred at EBM in Gothenburg, Sweden. The mice were immunized as described in Table 1, groups 1, 4, 5, and 7 and challenged with X-31 virus on day 41. Female BALB/c mice were used as wild-type (wt) controls. Clinical symptoms were assessed for 14 days using the scoring system described earlier.

### Sample Collection From Mice

Before sacrifice, blood was drawn by cheek puncture for determining IgG, IgA and neutralizing antibody titers. Nasal and lung washes were taken using 1 ml PBS (pH 7.4) with Complete® protease inhibitor cocktail (Roche, Almere, The Netherlands) for determining IgA titers. Lungs were collected in 1 ml complete EPISERF medium (100 U/ml penicillin, 100 mg/ml streptomycin, 12.5 ml of 1 M HEPES, 5 ml of 7.5% sodium bicarbonate for 500 ml medium, Thermo Fisher Scientific, Bleisweijk, Netherlands) for determination of viral load. Spleens were collected in 1 ml Iscove's Modified Dulbecco's Medium (IMDM) (Thermo Fisher Scientific, Bleisweijk, Netherlands) containing 10% v/v FBS (Lonza, Basel, Switzerland), 100 U/ml penicillin, 100 mg/ml streptomycin and 50 μM 2-mercaptoethanol (Invitrogen, Breda, The Netherlands) to assess cellular immune responses.

### Lung Virus Titration

Virus titration was performed as described previously (43). Briefly, the lungs were homogenized in 1 ml EPISERF medium and centrifuged at 1,200 rpm for 10 min to collect the supernatant. These supernatants were used to infect MDCK cells with serial 2 fold dilutions of the lung supernatants to determine lung virus titers as described before (43). Viral titers are presented as log_10_ titer of 50% tissue culture infectious dose per gram of lung. Limit of detection (LoD) was determined by calculating the log_10_ of the 1st dilution and the negative values were given half the value of the LoD.

### Assessment of Antibody Responses

Titers of influenza-specific IgG, IgG1, IgG2a, IgA, anti-NP, anti-M2e and neutralizing antibodies were determined in blood serum samples taken on day 41, i.e., the day of challenge. IgA was determined in mucosal samples immediately after sample collection. ELISAs were performed as described previously using WIV prepared from each of the challenge viruses, subunit vaccine (SU) prepared from X-31, NP protein, or M2e protein for coating (44). To determine whether the serum antibodies were (cross-)neutralizing, microneutralization (MN) assays were performed using infectious PR8, (H1N1)pdm09 or X-31 virus as described previously (45). LoD for IgG was determined by calculating the log_10_ of the 1st dilution while LoD for MN titers was calculated using Log_2_ of the 1st dilution.

### Multifunctional T Cell Assay.

To assess the contribution of influenza-specific T cells in protection, a multifunctional T cell assay was performed which involved staining for intracellular cytokines IFNγ, TNFα, IL2, and IL4 expressed by CD3+CD4+ and CD3+CD8+ T lymphocytes. All reagents, buffers and antibodies were purchased from eBioscience, The Netherlands.

Spleens collected in IMDM were immediately processed and single cell suspensions were obtained using GentleMACS C tubes and GentleMACS dissociator (Miltenyi Biotec, Leiden, The Netherlands). Cell suspensions were then forced through a cell strainer (BD Bioscience, Breda, The Netherlands) and erythrocytes lysed using ACK lysis buffer (0.83% NH_4_Cl, 10 mM KHCO_3_, 0.1 mM EDTA). Cells were re-stimulated with a final concentration of 10 μg/ml PR8, (H1N1)pdm09 or X-31 (H3N2) WIV plus 10 μg/ml of NP366 peptide, ASNENMETM for PR8, ASNENVETM for (H1N1)pdm09 or ASNENMDAM for X-31 (University Medical Center Leiden, The Netherlands) in the presence of co-stimulatory anti-CD28 antibody for 16 h. For each mouse, non-stimulated control cells were used to measure the baseline expression of the cytokines. After 12 h of incubation, protein transport inhibitor (Thermo Scientific, Netherlands) was added to stop the transport of proteins out of the Golgi apparatus. Cell stimulation cocktail containing PMA-ionomycin (Thermo Scientific, The Netherlands) was used as a positive control stimulant. Next day, cells were washed once with FACS buffer and stained for surface markers (anti-CD3-Alexa-fluor 700, anti-CD4-FITC, anti-CD8a PerCP-efluor720, all purchased from Thermo Scientific, The Netherlands) for 45 min at 4°C, followed by rinsing with cold PBS and staining with the fixable viability dye eFluor 780 for 30 min at 4°C. After two washes with FACS buffer, cells were fixed with fixation buffer and then permeabilized with FACS permeabilization buffer (Thermo Scientific). For intracellular cytokine staining (ICS), antibodies (anti-IFNγ-PE-Cy7, anti-IL2-PE, anti-TNFα eFluor 450 and anti-IL4 APC, Thermo Scientific) were added to the cells and incubated for 45 min. Ultracomp beads (Thermo Scientific, The Netherlands) were used to prepare compensation controls. Events were acquired on an LSRII flow cytometer (BD Biosciences) and FlowJo software (Tree Star) was used for analysis.

#### Statistics

For statistical analysis of intracellular cytokine levels, the numbers of cytokine positive and cytokine negative cells in the stimulated cell populations were compared with paired unstimulated controls using MIMOSA (Mixture Models for Single-Cell Assays) for IFNγ, TNFα, IL2 and IL4 (46). A false discovery rate of *q* ≤ 0.01 was accepted. A Chi-Squared test was used to compare the number of responders between groups. *P* ≤ 0.05 were considered significant.

The non-parametric Mann-Whitney U test was used to test if the differences between two groups with respect to different parameters were significant. A *p* < 0.05 was considered significant. Significance is represented as ^*^p < 0.05, ^**^p < 0.01, ^***^p < 0.0001. Statistical analyses were performed using GraphPad Prism version 5 for Windows (GraphPad Sofware, La Jolla, California, USA www.graphpad.com).

## Results

### WIV Combined With Mucosal Adjuvants Provides Best Cross-Protection

**Figure 1:**
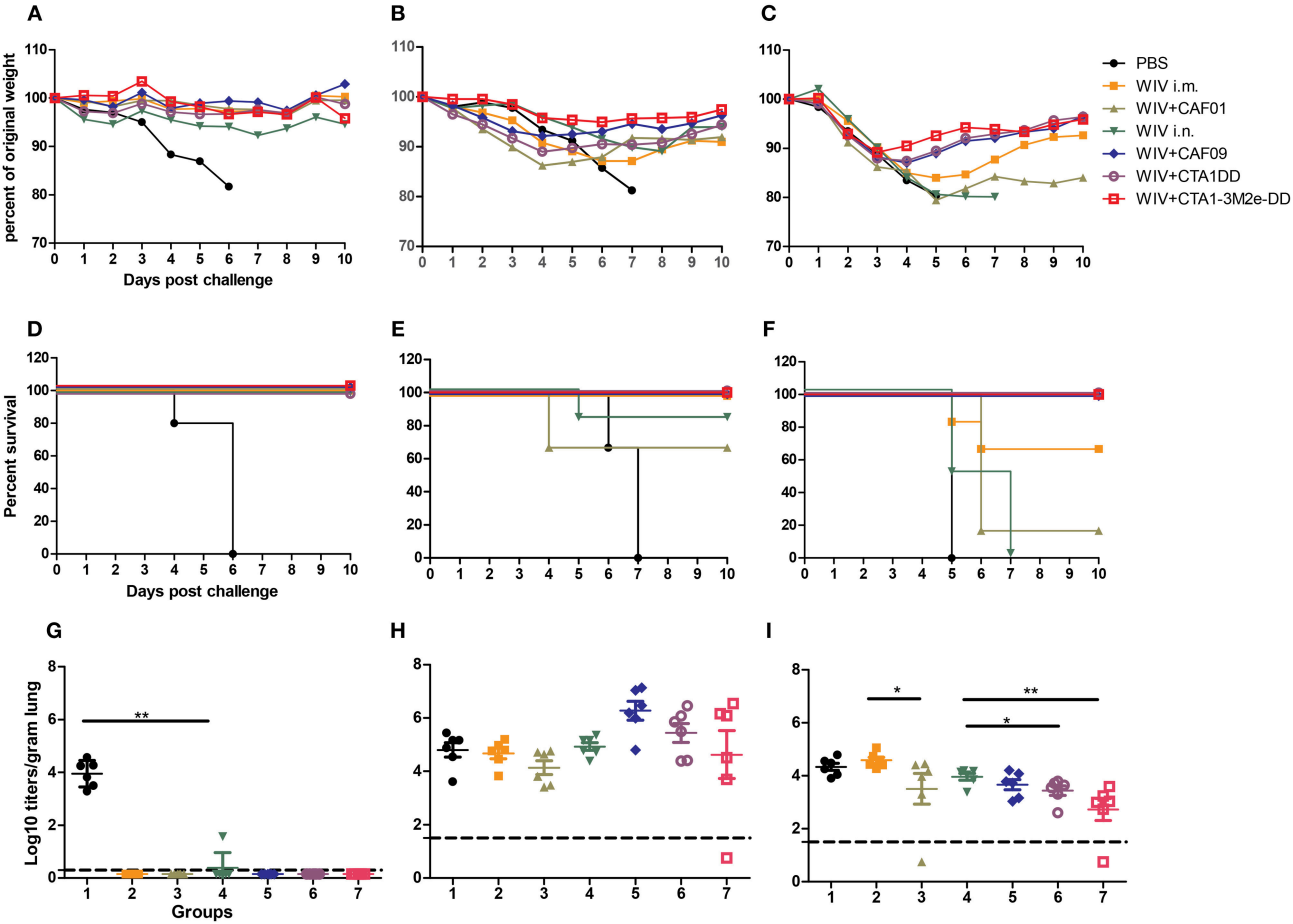
Adjuvanted i.n. administered vaccine provides best protection. CB6F1 mice were vaccinated thrice 10 days apart with PBS, non-adjuvanted or adjuvanted WIV vaccines. Three weeks after the last vaccination, 6 mice/group were challenged with homologous PR8 (H1N1) **(A,D,G)**, heterologous A/California/7/2009 (H1N1)pdm09 **(B,E,H)** or heterosubtypic X-31 (H3N2) viruses **(C,F,I)**. Six animals/group were followed for 10 days for weight loss **(A–C)** and survival **(D–F)**. Three days post challenge 6 mice/group were sacrificed for determining lung viral load **(G–I)**. Groups are represented as in Table 1: 1: PBS, 2: WIV i.m., 3: WIV+CAF01 i.m., 4: WIV i.n., 5: WIV+ CAF09 i.n., 6: WIV+ CTA1-DD i.n., 7: WIV+ CTA1-3Me-DD i.n. Dashed line indicates Limit of detection (LoD) (**G–I**). Virus titers are represented as log_10_ titers/gram of lung tissue with level of significance as ^*^p < 0.05 and ^**^p < 0.01 calculated using Mann-Whitney *U*-test.

In order to determine the relative efficacy of WIV vaccines combined with different adjuvants, mice were vaccinated three times via the most suitable route of administration with WIV derived from PR8 virus alone or mixed with CAF01, CAF09, CTA1-DD or CTA1-3M2e-DD, followed by challenge with homologous PR8, heterologous H1N1pdm09 (same virus subtype as PR8 but different strain) or heterosubtypic X-31 (different virus subtype but same internal viral proteins). To assess the protective efficacy of the tested vaccines, challenged mice were observed for weight loss and clinical symptoms for a period of 10 days or until they reached defined humane endpoint. Percent weight loss was calculated (Figures 1A–C) and survival curves were plotted (Figures 1D–F). Furthermore, to assess viral loads in the lungs, mock vaccinated and WIV vaccinated animals were sacrificed 3 days after homologous, heterologous or heterosubtypic virus challenge (Figures 1G–I).

When infected with PR/8 virus, all mock immunized mice reached the defined humane endpoint (>20% weight loss) and were sacrificed by day 6 post challenge. In contrast, mice from all groups immunized three times with WIV, with or without adjuvants, were protected from weight loss post challenge with homologous PR8 virus (Figures 1A,D). Furthermore, all but one (with low titer) of the vaccinated mice were completely protected from virus replication in the lungs (Figure 1G).

Challenge with heterologous (H1N1)pdm09 virus resulted in gradual weight loss in mock immunized animals gradually necessitating euthanasia on day 6 or 7 post infection. Animals vaccinated with non-adjuvanted WIV i.m. showed some weight loss but all survived until the end of the study. Surprisingly, mice immunized i.m. with CAF01 adjuvanted vaccine lost more weight and 2 out of 6 mice had to be sacrificed (Figures 1B,E). Animals vaccinated with WIV i.n. exhibited little weight loss except for one animal which reached the humane endpoint on day 5 post challenge. Mice vaccinated with WIV plus mucosal adjuvants presented the best cross-protection against heterologous virus challenge: they showed little or no weight loss and all animals survived to day 10 post challenge (Figures 1B,E). Although not significant, lung virus titers were somewhat higher in well protected than in unprotected, mock-immunized mice (Figure 1H).

In the heterosubtypic X-31 challenge experiment, all animals, whether vaccinated or not, initially showed a similar trend in weight loss (Figure 1C). However, from day 3 onwards, all the mice immunized mucosally with adjuvanted WIV recovered. In contrast, mock-immunized, parenterally immunized and mice immunized i.n. with WIV alone continued to lose weight and most animals had to be sacrificed, except for 4 out of 6 mice immunized i.m. with non-adjuvanted WIV (Figure 1F). Only mice mucosally immunized with adjuvanted WIV demonstrated significant reduction in lung viral titers as compared to mock-immunized control mice. CTA1-DD and CTA1-3M2e-DD adjuvanted vaccines afforded the largest reduction in lung viral titers (Figure 1I). Thus, i.n. immunization with CAF09, CTA1-DD or CTA1-3M2e-DD adjuvanted WIV stimulated significantly broader protection compared to systemic immunizations with WIV alone or WIV plus i.m. CAF01 adjuvant.

### Immuno-Profiling Reveals Strong Correlation Between Survival and Serum Antibodies, Mucosal IgA and IFNγ+ CD4 T Cells

**Figure 2:**
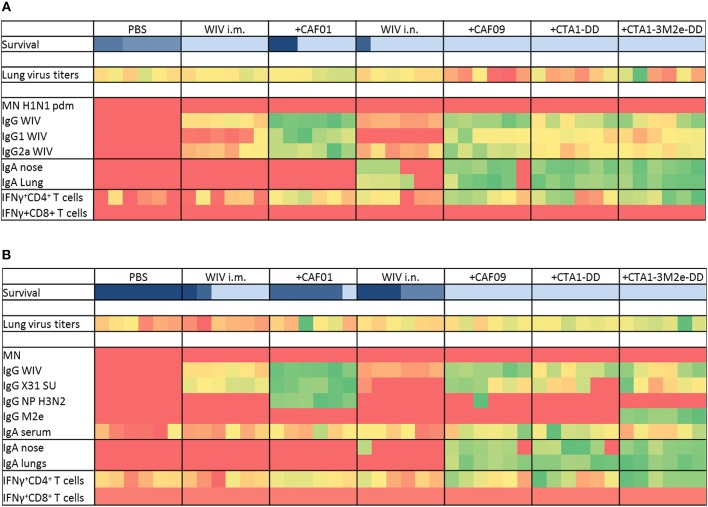
Immunoprofiling against heterologous and heterosubtypic viruses. Animals were vaccinated 3 times with the vaccines indicated in Table 1. After 3 vaccinations, sera, nasal, and lung washes and spleens were collected to determine systemic, mucosal and cell mediated immune responses (*n* = 6). Some animals were challenged with heterologous **(A)** and heterosubtypic **(B)** virus to determine protection, lung viral load (*n* = 6) and survival (*n* = 6). Generated data was used as input and conditional formatting was performed in Ms Excel to plot heatmaps. Each column represents one animal. Survival is shown with different color scheme as these are different animals compared to the rest. Dark blue indicates worst survival while light blue indicates best survival. For other parameters, heatmaps range from red (lowest response) to green (best response).

To determine which immune mechanisms correlated with the observed cross-protection and to what degree these mechanisms would differ for the different adjuvanted vaccines, various immunological assays were performed. Sera, nasal and lung washes were collected 3 weeks after the 3rd immunization for antibody titer assessments, while T cell responses against heterologous (H1N1)pdm09 and heterosubtypic X-31 virus were determined using spleens of vaccinated animals 3 days post heterosubtypic challenge. The results of immunoprofiling for the heterologous and heterosubtypic challenge experiments are summarized as heatmaps (Figures 2A,B) to reveal patterns which correlate with protection; the individual data can be found in the Supplementary Information (Figures S1–S5).

Immunization with PR8 WIV i.m. reliably induced neutralizing antibodies against the homologous virus, especially when administered with adjuvant (Figure S1A). By contrast, i.n. immunizations poorly stimulated neutralizing antibodies, even in the presence of adjuvants. Importantly, we found no neutralizing antibodies against heterologous (H1N1)pdm09 or heterosubtypic X-31 virus irrespective of the immunization route or adjuvant used (Figures 2A,B and Figures S1B,C). However, all immunized mice developed serum IgG antibodies reactive with homologous, as well as heterologous and heterosubtypic virus and these titers were of identical magnitude for all three virus strains (Figures 2A,B and Figures S1D–F). The addition of adjuvants to i.m. and i.n WIV immunizations enhanced cross-reactive serum IgG resulting in similar endpoint titers. CAF01 and CAF09 affected IgG titers most strongly and enhanced both IgG1 and IgG2a. CTA1-DD and CTA1-3M2e-DD were comparatively less effective in stimulating IgG and IgG1 and had only minor effects on IgG2a levels (Figures 2A,B and Figures S2A,B).

Furthermore, we wanted to identify the antigens targeted by the cross-reactive IgG. Use of subunit vaccine for coating revealed that vaccine-evoked, cross-reactive antibodies readily bound to viral surface proteins. These antibodies were found in all mice immunized i.m., but were present in mice immunized i.n. only when adjuvanted vaccine was used (Figures 2A,B, Figure S2C). Anti-NP antibodies were detected only in mice vaccinated with WIV plus CAF01 and one mouse from the WIV plus CAF09 group (Figures 2A,B and Figure S2D). Anti-M2e antibodies were induced only by WIV adjuvanted with CTA1-3M2e-DD (Figures 2A,B and Figure S2E). Vaccination, especially when done with adjuvanted vaccines, therefore induced cross-reactive antibodies which mainly targeted the viral surface proteins. The levels of these antibodies correlated with protection from severe disease, except in the group with CAF01-adjuvanted vaccine delivered i.m.

Determination of influenza specific mucosal IgA revealed that mice from the PBS control group as well as mice immunized i.m. with non-adjuvanted or CAF01-adjuvanted WIV developed no or very low mucosal IgA responses (in nose and lungs) against any of the viruses (Figures 2A,B and Figures S3A,B,C). In contrast, all miceimmunized i.n. with adjuvanted WIV produced significant levels of specific IgA antibodies in both nose and lungs against all three virus strains, and these levels were significantly higher than in mice immunized i.n. with non-adjuvanted WIV. Therefore, mucosal immunization in the presence of adjuvant was required for successful induction of cross-reactive mucosal IgA (Figures 2A,B and Figures S3A,B,C). IgA titers strongly correlated with protection from weight loss (Figures 2A,B).

We next assessed vaccine-induced T cell responses. *In vitro* re-stimulation of splenocytes with heterologous or heterosubtypic WIV demonstrated that mice immunized i.m and mice immunized i.n. with non-adjuvanted WIV developed no or very low levels of IFNγ-producing CD4 T cells. Mice immunized i.n. with adjuvanted WIV demonstrated enhancement of IFNγ-producing cells, with CTA1-3M2e-DD being most potent. In addition to IFNγ, we also measured IL2 and TNFα responses in CD4+ T cells, but although restimulation with WIV and peptides increased the numbers of T cells producing these cytokines, the percentages were low and no significant differences between immunized and mock-immunized animals were observed (results not shown). The large majority of vaccine-specific CD4 T cells produced IFNγ, while very few cells were multifunctional, also producing other cytokines (Figure S5). In contrast to CD4+ T cells, CD8+ T cells were not induced in significant numbers by any of the vaccines (Figures 2A,B and Figures S4C,D). In conclusion, IFNγ-producing CD4 T cells were the only T cell population induced and their numbers were enhanced significantly by adjuvanted WIV administered i.n. Protection from weight loss correlated well with the number of IFNγ-producing CD4 T cells (Figures 2A,B and Figures S4A,B).

### Dissecting the Mechanisms of Protection

From the heat maps it can be deduced that animals which were completely protected from heterosubtypic challenge had high levels of serum IgG and mucosal IgA and high numbers of IFNγ-producing CD4 T cells. We next performed a series of experiments in order to determine whether any of these factors was critical for protection from heterosubtypic virus infection. For these experiments we focused on CAF09 and CTA1-3M2e-DD as the most successful adjuvants from the previous experiment and used PBS and non-adjuvanted WIV as controls.

**Figure 3:**
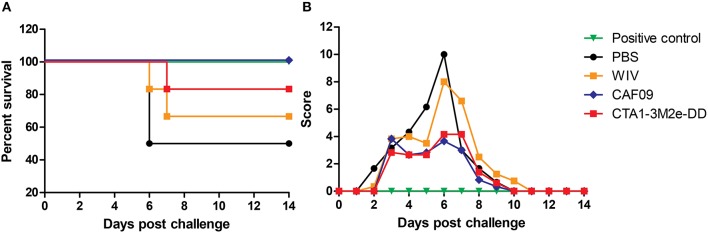
Serum antibodies induced by mucosally adjuvanted WIV might induce cross-protection. Serum was collected from animals vaccinated thrice with PBS i.n., non-adjuvanted WIV i.n., WIV+ CAF09 i.n., or WIV+ CTA1-3M2e-DD i.n. and was administered passively in naïve mice (*n* = 6) via the i.p. route. One day after passive immunization animals were challenged with heterosubtypic X-31 virus. Animals from the positive control group received PR8 immune sera and were challenged with homologous PR8 virus. The mice were followed for survival **(A)** and clinical symptoms **(B)** assessed using a score based on weight, appearance and behavior.

To assess if serum antibodies can mediate cross-protection, mice were passively immunized via the i.p. route with serum collected from animals which had been mock-immunized with PBS or immunized with WIV, WIV+CAF09 or WIV+CTA1-3M2e-DD i.n. One day later they were challenged with heterosubtypic X-31 virus. Animals which received PR8 immune serum followed by a homologous challenge with PR8 virus served as positive control group. Mice were observed daily for clinical symptoms using the score sheet described previously. We found that PR8 immune serum completely protected mice against PR8 virus infection. Serum from mice immunized i.n. with PR8 WIV without adjuvant did not provide protection against infection with heterosubtypic X-31 virus and the mice transfused with this serum exhibited high clinical scores and reduced survival (Figures 3A,B). By contrast, serum from mice immunized with WIV plus CAF09 or CTA1-3M2e-DD protected partially and clinical scores were reduced by 50% compared to unimmunized control mice. Thus, non-neutralizing serum IgG antibodies from mice immunized i.n. with CAF09 or CTA1-3M2e-DD adjuvanted WIV appeared to partially protect against heterosubtypic X-31 virus challenge.

**Figure 4:**
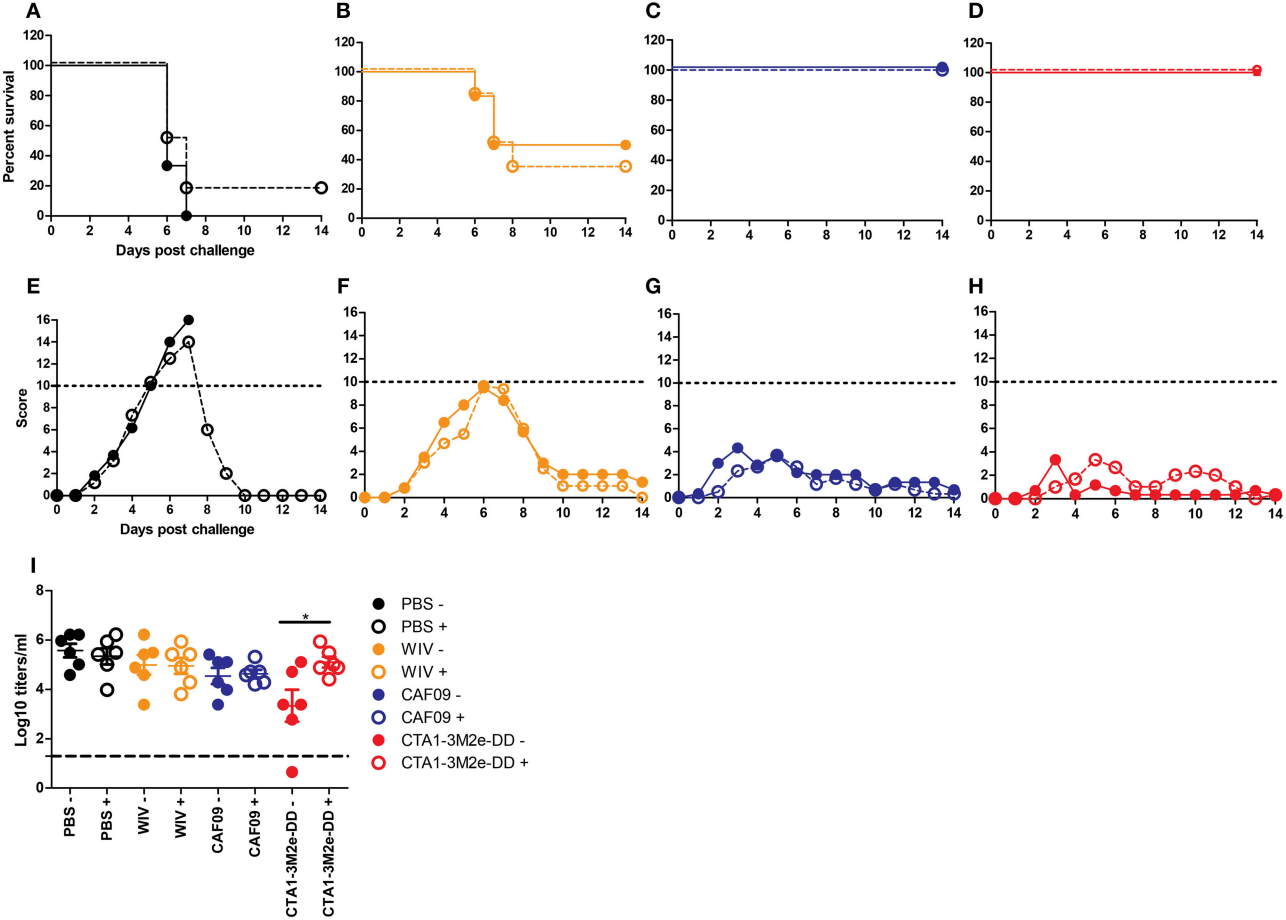
CD4 depletion does not affect protection but affects virus growth. Mice (*n* = 12/group) were vaccinated thrice i.n. with PBS **(A,E)**, non-adjuvanted WIV **(B,F)**, WIV+ CAF09 **(C,G)** or WIV+ CTA1-3M2e-DD **(D,H)** followed by heterosubtypic challenge. On day−1, 1 and 7 relative to the challenge, anti-CD4 antibody or PBS was administered i.p.. Mice were followed for 14 days for survival **(A–D)** and clinical symptoms **(E–H)**. Dotted lines in E-H indicate the humane endpoint. Six animals/group were sacrificed on day 3 post challenge to determine lung virus titers **(I)**. LoD is indicated by dashed line. Mock depletion is presented by filled symbols with—and CD4 depletion is represented by open symbols with +. Virus titers are represented as log_10_ titers /gram of lung with level of significance as ^*^p < 0.05 calculated using Mann-Whitney *U*-test.

Next, we studied the role of CD4+ T cells in protection. Depletion of CD4 T cells was achieved through anti-CD4 Mab-treatment of mice and resulted in reduction of CD4 T cell numbers in peripheral blood by >95% (data not shown). We found that CD4 depletion did not affect survival (Figures 4A–D) or clinical scores (Figures 4E–H) upon X-31 virus infection in the well immunized animals. Yet, CD4 T cell depletion had a significant effect on lung virus titers in animals immunized with WIV and CTA1-3M2e-DD while all other immunization protocols showed comparable lung virus titers irrespective of CD4 depletion (Figure 4I). Therefore, CD4 T cells appeared to play a role in protection against heterobsubtypic challenge only in the CTA1-3M2e-DD group.

**Figure 5:**
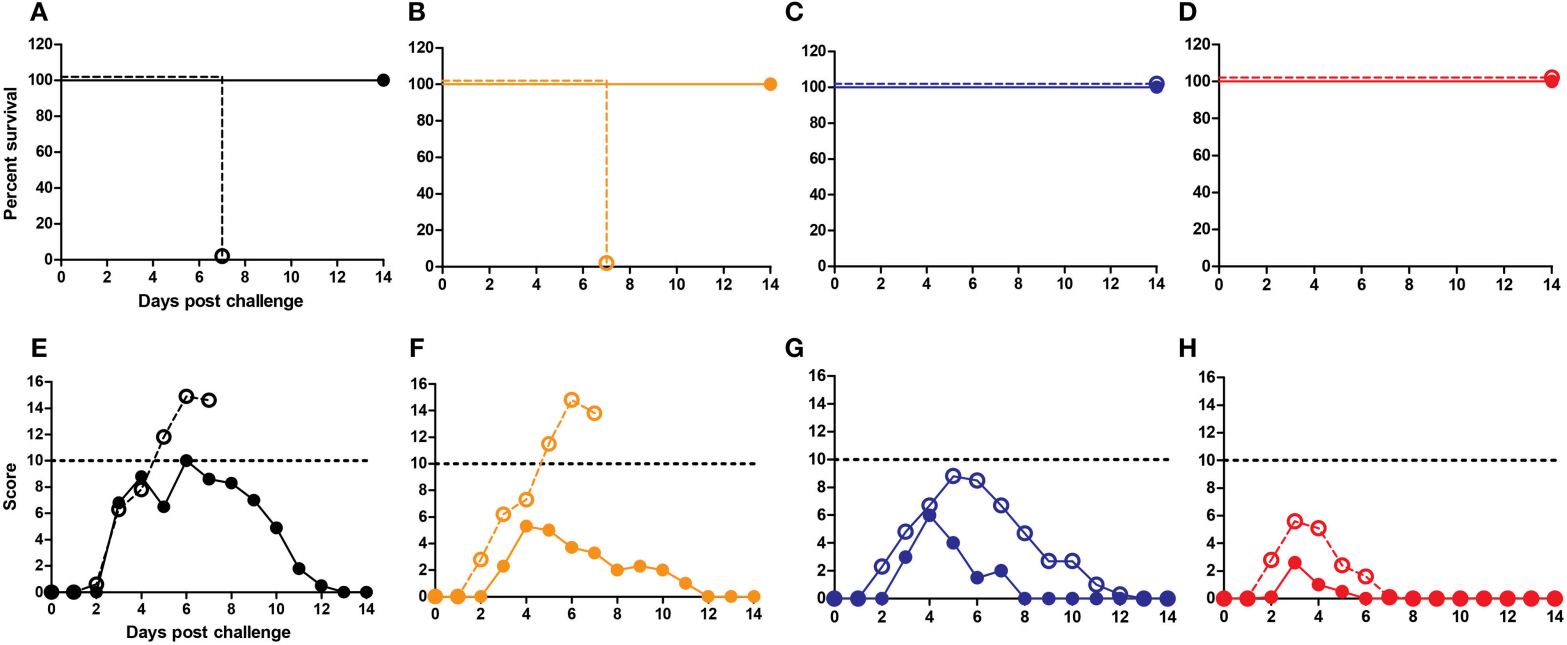
IgA antibodies are not critical in cross-protection. IgA KO mice and BALB/c mice were vaccinated thrice i.n. with PBS **(A,E)**, non-adjuvanted WIV **(B,F)**, WIV+CAF-09 **(C,G)** or WIV+CTA1-3M2e-DD **(D, H)** followed by heterosubtypic challenge. The mice were followed for survival **(A–D)** and development of clinical symptoms **(E–H)** for a period of 14 days. IgA KO mice are represented by dashed lines with open symbols while BALB/c wt mice are represented by solid lines with filled symbols. Dotted lines indicate humane endpoint.

Finally, we addressed whether cross-reactive local IgA antibodies impacted on protection against infection in the mice immunized i.n. by repeating the immunization/challenge experiment in IgA KO mice. In line with reports in literature, we found that mock-immunized IgA KO mice were more susceptible to influenza infection than mock-immunized wt BALB/c mice, demonstrated by higher clinical scores and survival post challenge (47). Wild-type BALB/c mice immunized with non-adjuvanted WIV demonstrated reduced clinical scores as compared to non-immunized BALB/c mice but this was not the case for IgA KO mice indicating a role for IgA in protection (Figures 5A,E and Figures 5B,F). When immunized with WIV and any of the mucosal adjuvants, wt and IgA KO mice developed protective immunity and survived the challenge infection (Figures 5C,D,G,H). Clinical scores of IgA KO mice immunized with CAF09 adjuvanted vaccine were higher than those of wt mice (Figure 5G). Mice immunized with CTA1-3M2e-DD adjuvanted WIV developed the lowest clinical scores with little difference between wt and IgA KO mice (Figure 5H). These results suggest that local IgA antibodies exerted some protection from severe disease but were not critical for survival in this model.

## Discussion

In this study, we compared liposome and protein adjuvants head-to-head to assess their relative efficacy in inducing cross-reactive immunity in mice, when combined with i.m. and i.n. administered WIV. In addition, we dissected which immune parameters contributed to protection and to what extent these would be vaccine-specific. The results indicate that i.n. administered WIV combined with a mucosal adjuvant provided enhanced cross-protection compared to WIV administered i.m. with or without adjuvant and non-adjuvanted WIV administered i.n. We observed that non-neutralizing serum IgG, mucosal IgA and IFNγ-producing CD4 T cells were significantly higher for mice immunized i.n. with WIV plus adjuvant than for the other, less well protected groups. While non-neutralizing serum IgG antibodies and CD4 T cells were contributing to protection, our experiments in IgA KO mice were less conclusive, but there was a trend toward a protective effect of local IgA on the clinical symptoms.

Mucosal immunization has been shown to be superior to parenteral immunization for stimulating local immunity and resident memory T cells in the lung (48–50) and to provide cross-protection against heterosubtypic virus challenge (14, 51). In agreement with these studies, we found that i.n. immunization with adjuvanted WIV afforded stronger cross-protection than parenteral immunizations. This was the case even though serum anti-viral IgG levels appeared quite comparable for mice immunized i.m. or i.n. with adjuvanted vaccines. Upon heterologous infection with (H1N1)pdm09 virus, clinical symptoms and survival correlated poorly with virus replication in the lungs while for heterosubtypic infection with X-31 virus we observed a clear correlation between clinical scores and lung virus titers. We chose day 3 to assess the lung viral load because we wanted to check if the vaccination has any impact on the replicating virus at a stage where the replication is at peak which in mice is on day 3 (52, 53). From experience we assume that in successfully immunized mice virus titers resolved more quickly than in mock immunized mice (45).

Adjuvanted WIV vaccines induced significantly higher systemic immune responses compared to non-adjuvanted WIV. Interestingly, the levels of serum IgG antibodies reacting with homologous, heterologous and heterosubtypic WIV in ELISA assays were similar, suggesting that most of the IgG antibodies induced by immunization with WIV were cross-reactive. This is in line with recent observations in humans that also indicate that many influenza-specific antibodies, whether measured before or after vaccination, are cross-reactive (54, 55). Although serum antibodies induced by adjuvanted WIV were cross-reactive they could not neutralize heterologous and heterosubtypic virus *in vitro*, which was according to expectations (45, 56). Nevertheless, when transferred to naïve animals these antibodies provided partial protection against X-31 challenge. It has been shown that anti-influenza antibodies can mediate cross-protection via non-neutralizing mechanisms such as antibody dependent cellular cytotoxicity (ADCC), antibody dependent cellular phagocytosis, or complement mediated cytotoxicity (CDC) (57–60). Which of these mechanisms, if any, is involved in protection afforded by WIV adjuvanted with CAF09 or CTA1-3M2e-DD remains to be elucidated.

Vaccination with WIV plus mucosal adjuvants also led to remarkably enhanced levels of cross-reactive local IgA in the lungs and nose of mice. IgA has been shown to be more cross-reactive than IgG (61, 62). Moreover, Maurer et al have recently shown that IgA antibodies can also neutralize influenza virus in an antigen-aspecific manner by providing sialic acid in the glycosylated Fc part which serves as a decoy receptor not only for influenza virus but also for other viruses using sialic acid as a receptor (63). However, in our study adjuvanted WIV administered i.n. completely protected IgA KO mice from reaching the humane endpoint post heterosubtypic challenge, similar to wt BALB/c mice, indicating that IgA was not crucial for protection. Nevertheless, we cannot exclude that local IgA antibodies exerted some protective function, as reflected in the higher clinical scores in immunized and challenged IgA KO mice compared to the wt mice. Whether local IgA plays a role in cross-protection induced by adjuvanted WIV administered i.n. or not is controversial. For example, Zhang et al. (64) showed that i.n. immunization with a CTB/CT-adjuvanted subunit vaccine stimulated equally efficient control of virus growth in wt and IgA KO mice. Yet, using mice deficient in the poly Ig receptor and thus unable to transport IgA across the respiratory epithelium, Asahi et al demonstrated that mucosal IgA is critical, in particular for protection from heterologous virus challenge (65). From our observations, we think that mucosal IgA, though not being crucial on its own, works in concert with other mechanisms to provide the observed cross-protection.

IFNγ-producing cross-reactive CD4 positive T cells increased significantly upon mucosal vaccination, especially in mice immunized with WIV plus CTA1-3M2e-DD. This could be due to the fact that M2e contains an MHC class II restricted CD4 helper epitope (26). CD4 depletion shortly before and after challenge did not affect survival, but impaired the control of lung virus growth in animals vaccinated with WIV plus CTA1-3M2e-DD, indicating a role of CD4 T cells in controlling virus growth rather than controlling clinical symptoms and in turn survival. CAF09-adjuvanted WIV did not induce significant numbers of CD4 T cells and accordingly, depletion of CD4 T cells during infection did not affect clinical scores or lung virus titers.

We did not observe IFNγ-producing CD8 T cells induced by vaccination. This was somewhat unexpected since at least CAF01, CAF09, and CTA1-DD are known to support induction of CD8 T cells (24, 33, 37, 66). Failure to detect CD8 T cells in our experiments might be because they were indeed absent or because we missed them due to the timing of the experiments. We assessed CD8 T cell responses on day 25 after the last immunization, by which time the cells were already through the retraction phase and the numbers might therefore have been too low to detect. Furthermore, we used intracellular cytokine staining, a method which allows simultanous determination of multiple cell populations and cytokines but is not as sensitive as ELISPOT assay for the detection of CD8 T cells. Despite the fact that we did not find them, we cannot categorically rule out that CD8 T cells contributed to protection. It is known that lung tissue resident memory (TRM) CD8 T cells have an important role in protection from influenza infection (66, 67). These cells might indeed have been induced in our experiments, but as parts of the lungs had to be used for other purposes and the remaining tissues were insufficient for isolating the required numbers of lymphocytes, we were unable to investigate them. Follow up studies will address this issue.

Although WIV plus CAF01 induced the highest levels of cross-reactive antibodies, mice in the respective experimental group showed severe clinical symptoms and reduced survival post challenge. One thing which distinguished the CAF01 vaccinated mice group from the other groups was the induction of anti-NP antibodies. In a pre-clinical study in pigs by Ricklin et al. NP vaccination produced a strong immune response but induced lung inflammation and immunized pigs were not protected (68). In murine models the situation is not entirely clear. Although it has been shown that anti-NP antibodies can confer resistance to influenza virus infection (69–72) (contrary to our results), a previous study from our group demonstrated that mice vaccinated with NP adjuvanted with MPLA (which developed NP-specific antibodies) showed more rapid weight loss in the initial phase of infection than mock vaccinated mice (73). Furthermore, animals receiving a virosomal vaccine without NP showed fewer signs of illness compared to mice receiving a virosomal vaccine with NP.

One of the limitations of the study is the use of X-31 virus for heterosubtypic challeng. X-31 is a ressortant between PR8 and A/HK/68 and thus contains internal genes derived from PR8 virus. In future studies it would be ideal to use a wild type H3N2 virus for challenge and further also performing challenge experiments with avian influenza viruses to assess the breadth of protection afforded by the adjuvanted vaccines. Another limitation is the fact that heterologous virus challenge was performed 3 weeks after the last immunization when adjuvant-induced innate immune responses might still have been present. However, for CAF09 little induction of (systemic) innate immunity has been observed (Christensen et al. unpublished observations) while for CTA1-DD a low level of antigen-independent protection was observed independent of the time period (2, 4, or 6 weeks) between immunization and challenge (Lycke et al. unpublished observations). Thus, while we cannot completely rule out an effect of adjuvant-induced innate responses we consider it unlikely that these are the major reason for the observed cross-protection. Future experiments devoted to assessing the durability of the induced immune responses by increasing the period between immunization and challenge will clarify this point definitely.

The results of our head-to-head comparison of different vaccines underline that mucosal immunization with adjuvanted WIV is indeed a promising approach for developing a broadly protective influenza vaccine. These vaccines induce a plethora of immune responses including mucosal IgA, cross-reactive (though not cross-neutralizing) systemic IgG, and CD4 Th cells. Each of these seems to play a role in cross-protection but neither appeared to be crucial. This indicates that, several immune mechanisms contribute to the cross-protection induced by mucosal vaccination with adjuvanted WIV and optimal protection thus requires a combination of different mechanisms.

## Author Contributions

YB, AH, NL, and DC designed the study. YB, WD, IG, and TM performed the animal experiments. YB, JdV-I, DV, and SN performed assays and analysis of data. KG, SS, and OE produced live and inactivated viruses and characterized them. LB provided CD4 depletion antibody. YB and AH wrote the manuscript.

### Conflict of Interest Statement

LB is employed by Bioceros. The remaining authors declare that the research was conducted in the absence of any commercial or financial relationships that could be construed as a potential conflict of interest.
