# Supplementary material for: Cross-Protective Potential and Protection-Relevant Immune Mechanisms of Whole Inactivated Influenza Virus Vaccines Are Determined by Adjuvants and Route of Immunization
Source: Front Immunol. 2019 Mar 29;10:646. doi: 10.3389/fimmu.2019.00646 (PMC6450434; doi:10.3389/fimmu.2019.00646)
Supplement: Supplementary file 1 [file Presentation_1.PPTX]

## Slide 1
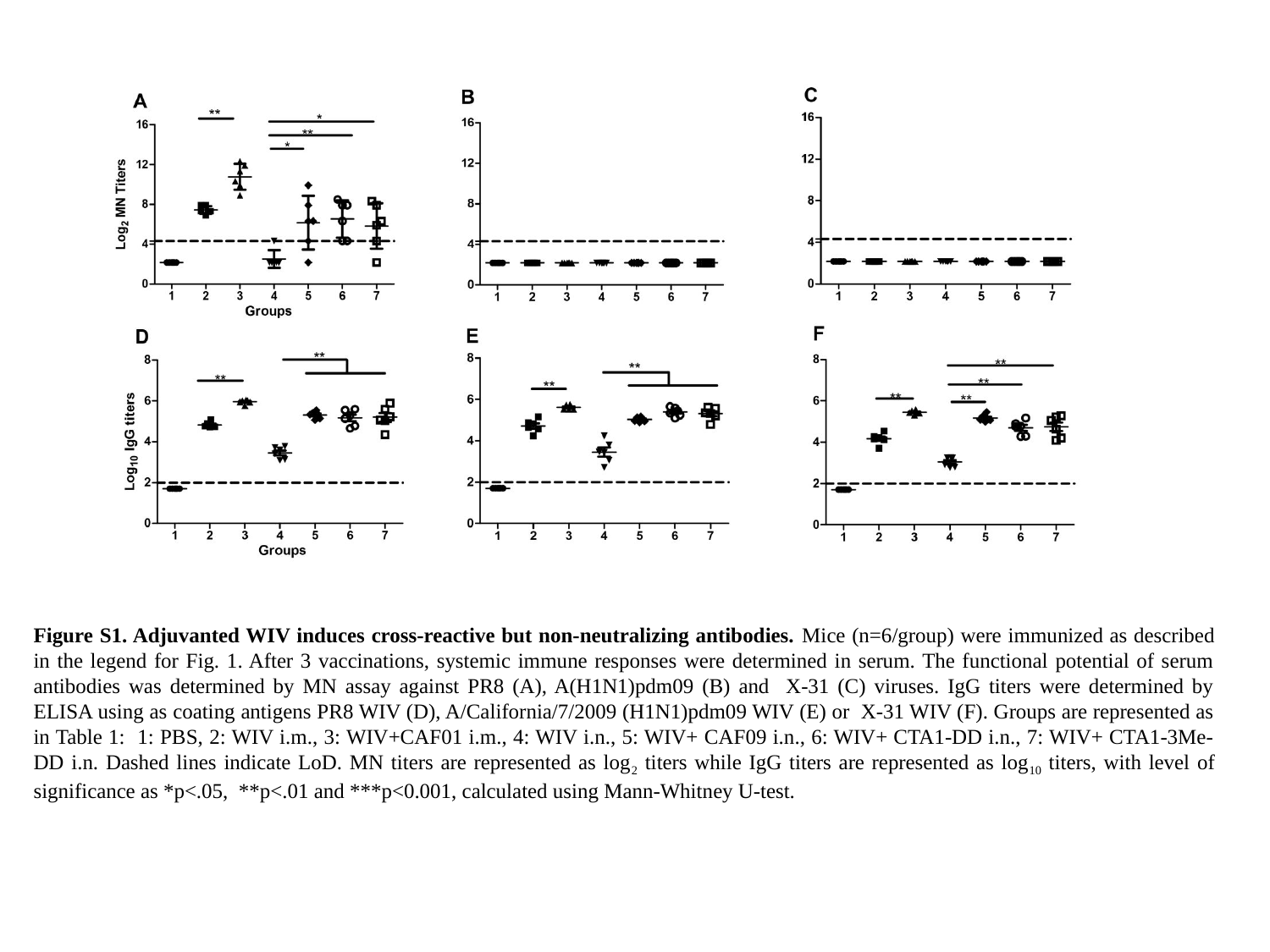

Figure S1. Adjuvanted WIV induces cross-reactive but non-neutralizing antibodies. Mice (n=6/group) were immunized as described in the legend for Fig. 1. After 3 vaccinations, systemic immune responses were determined in serum. The functional potential of serum antibodies was determined by MN assay against PR8 (A), A(H1N1)pdm09 (B) and X-31 (C) viruses. IgG titers were determined by ELISA using as coating antigens PR8 WIV (D), A/California/7/2009 (H1N1)pdm09 WIV (E) or X-31 WIV (F). Groups are represented as in Table 1: 1: PBS, 2: WIV i.m., 3: WIV+CAF01 i.m., 4: WIV i.n., 5: WIV+ CAF09 i.n., 6: WIV+ CTA1-DD i.n., 7: WIV+ CTA1-3Me-DD i.n. Dashed lines indicate LoD. MN titers are represented as log2 titers while IgG titers are represented as log10 titers, with level of significance as *p<.05, **p<.01 and ***p<0.001, calculated using Mann-Whitney U-test.
