# Supplementary material for: Cross-Protective Potential and Protection-Relevant Immune Mechanisms of Whole Inactivated Influenza Virus Vaccines Are Determined by Adjuvants and Route of Immunization
Source: Front Immunol. 2019 Mar 29;10:646. doi: 10.3389/fimmu.2019.00646 (PMC6450434; doi:10.3389/fimmu.2019.00646)
Supplement: Supplementary file 2 [file Presentation_2.pptx]

## Slide 1
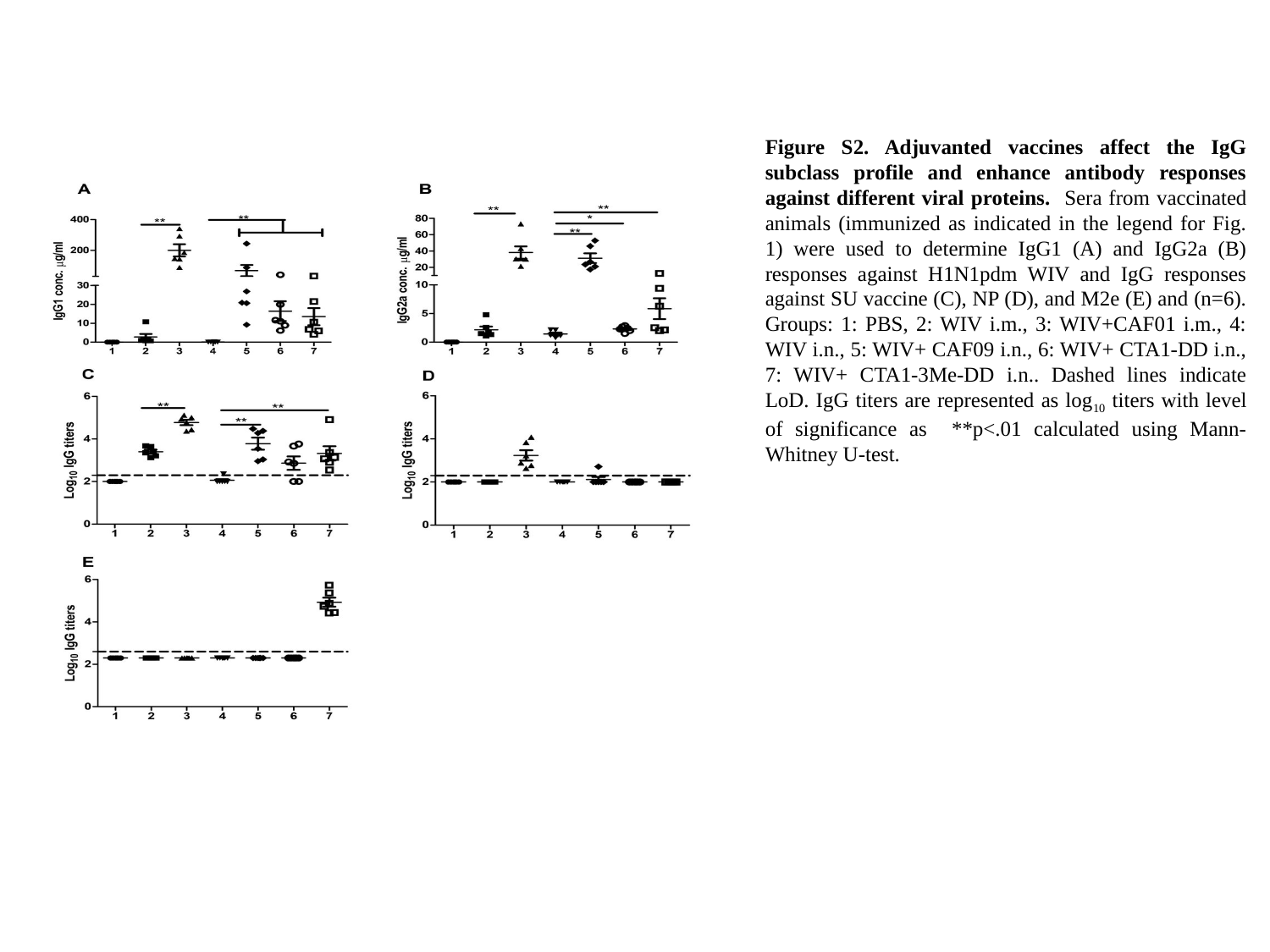

Figure S2. Adjuvanted vaccines affect the IgG subclass profile and enhance antibody responses against different viral proteins. Sera from vaccinated animals (immunized as indicated in the legend for Fig. 1) were used to determine IgG1 (A) and IgG2a (B) responses against H1N1pdm WIV and IgG responses against SU vaccine (C), NP (D), and M2e (E) and (n=6). Groups: 1: PBS, 2: WIV i.m., 3: WIV+CAF01 i.m., 4: WIV i.n., 5: WIV+ CAF09 i.n., 6: WIV+ CTA1-DD i.n., 7: WIV+ CTA1-3Me-DD i.n.. Dashed lines indicate LoD. IgG titers are represented as log10 titers with level of significance as **p<.01 calculated using Mann-Whitney U-test.
