# Supplementary material for: Cross-Protective Potential and Protection-Relevant Immune Mechanisms of Whole Inactivated Influenza Virus Vaccines Are Determined by Adjuvants and Route of Immunization
Source: Front Immunol. 2019 Mar 29;10:646. doi: 10.3389/fimmu.2019.00646 (PMC6450434; doi:10.3389/fimmu.2019.00646)
Supplement: Supplementary file 3 [file Presentation_3.PPTX]

## Slide 1
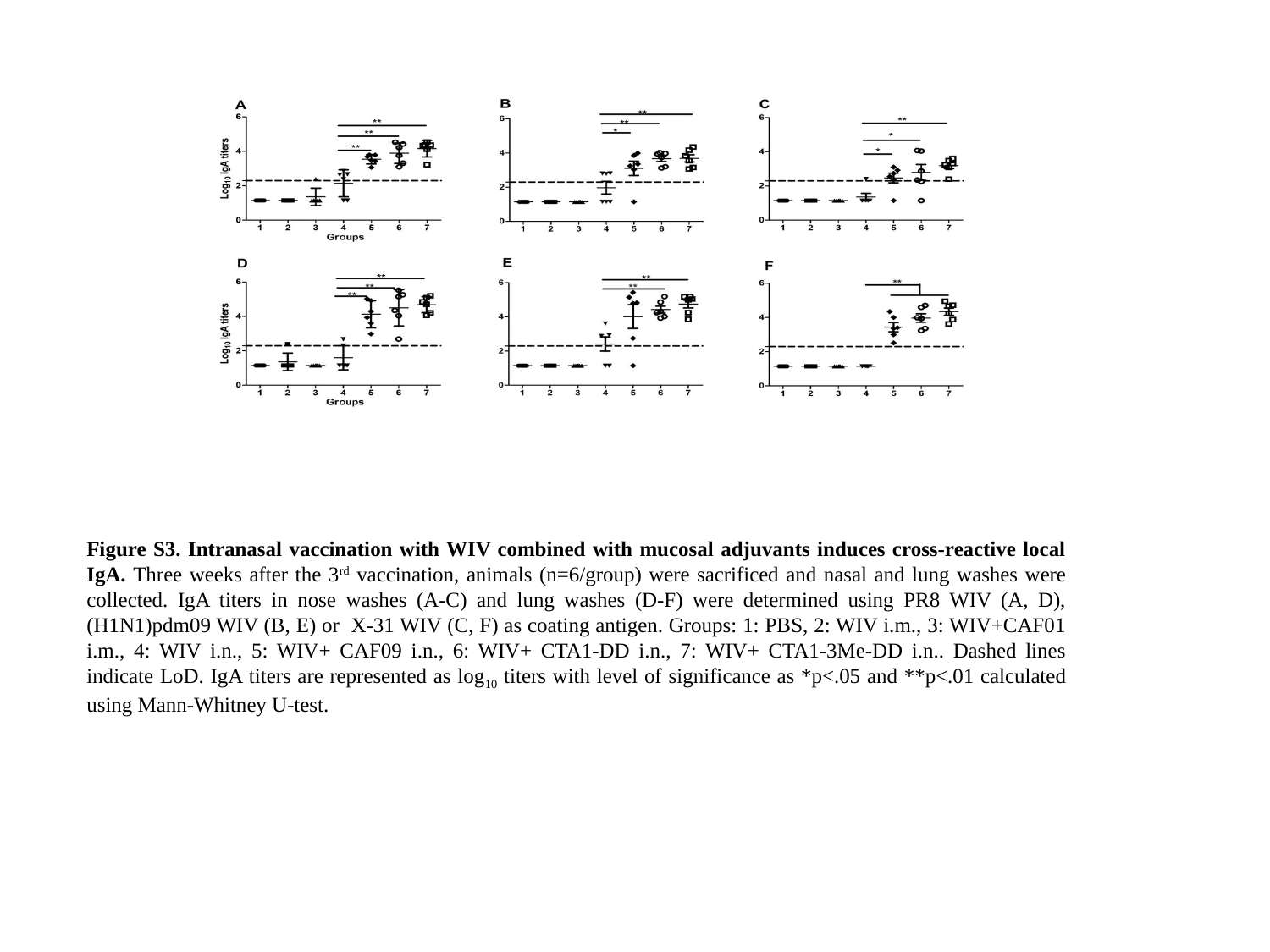

Figure S3. Intranasal vaccination with WIV combined with mucosal adjuvants induces cross-reactive local IgA. Three weeks after the 3rd vaccination, animals (n=6/group) were sacrificed and nasal and lung washes were collected. IgA titers in nose washes (A-C) and lung washes (D-F) were determined using PR8 WIV (A, D), (H1N1)pdm09 WIV (B, E) or X-31 WIV (C, F) as coating antigen. Groups: 1: PBS, 2: WIV i.m., 3: WIV+CAF01 i.m., 4: WIV i.n., 5: WIV+ CAF09 i.n., 6: WIV+ CTA1-DD i.n., 7: WIV+ CTA1-3Me-DD i.n.. Dashed lines indicate LoD. IgA titers are represented as log10 titers with level of significance as *p<.05 and **p<.01 calculated using Mann-Whitney U-test.
