# Supplementary material for: Cross-Protective Potential and Protection-Relevant Immune Mechanisms of Whole Inactivated Influenza Virus Vaccines Are Determined by Adjuvants and Route of Immunization
Source: Front Immunol. 2019 Mar 29;10:646. doi: 10.3389/fimmu.2019.00646 (PMC6450434; doi:10.3389/fimmu.2019.00646)
Supplement: Supplementary file 4 [file Presentation_4.PPTX]

## Slide 1
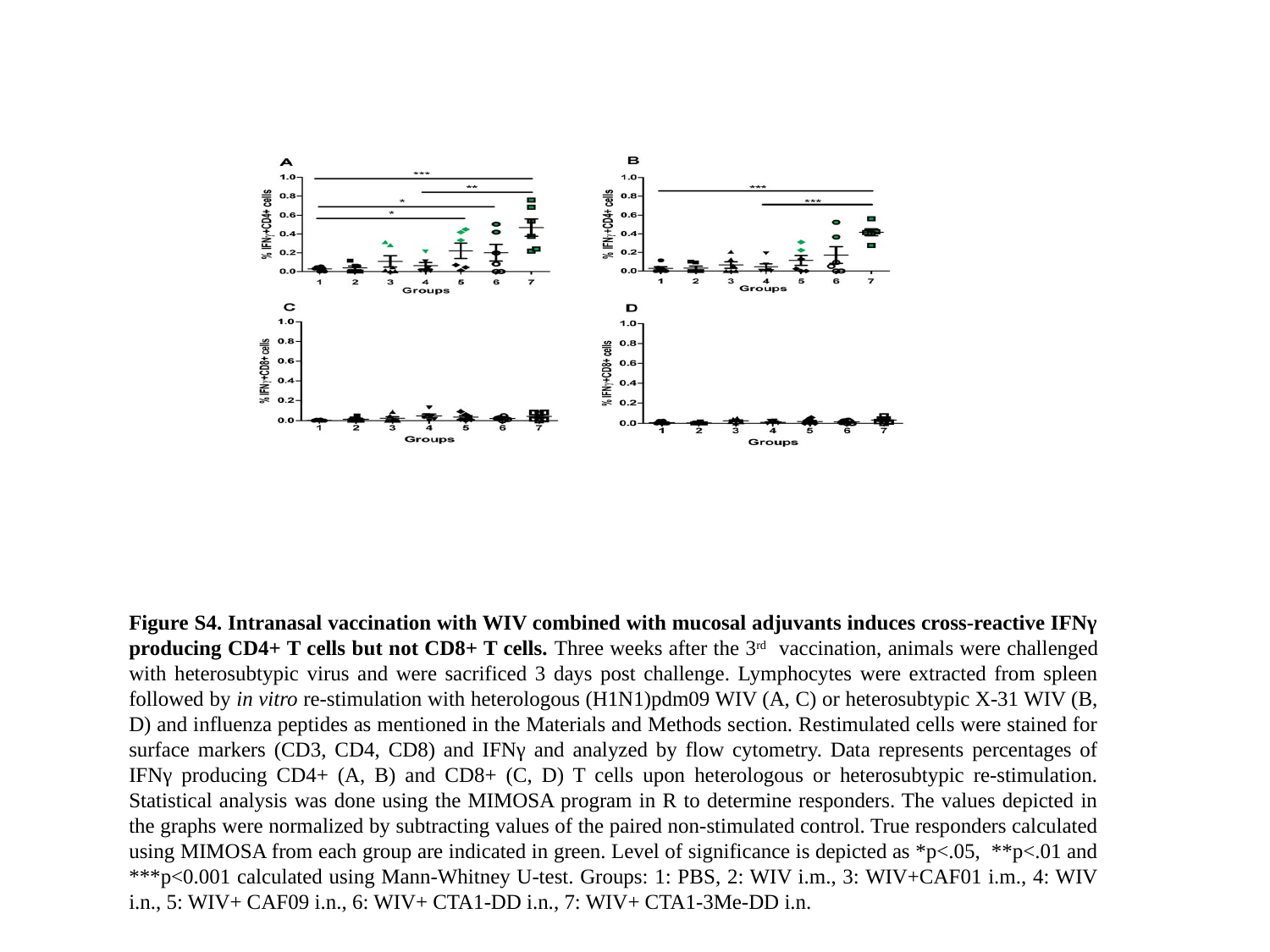

Figure S4. Intranasal vaccination with WIV combined with mucosal adjuvants induces cross-reactive IFNγ producing CD4+ T cells but not CD8+ T cells. Three weeks after the 3rd vaccination, animals were challenged with heterosubtypic virus and were sacrificed 3 days post challenge. Lymphocytes were extracted from spleen followed by in vitro re-stimulation with heterologous (H1N1)pdm09 WIV (A, C) or heterosubtypic X-31 WIV (B, D) and influenza peptides as mentioned in the Materials and Methods section. Restimulated cells were stained for surface markers (CD3, CD4, CD8) and IFNγ and analyzed by flow cytometry. Data represents percentages of IFNγ producing CD4+ (A, B) and CD8+ (C, D) T cells upon heterologous or heterosubtypic re-stimulation. Statistical analysis was done using the MIMOSA program in R to determine responders. The values depicted in the graphs were normalized by subtracting values of the paired non-stimulated control. True responders calculated using MIMOSA from each group are indicated in green. Level of significance is depicted as *p<.05, **p<.01 and ***p<0.001 calculated using Mann-Whitney U-test. Groups: 1: PBS, 2: WIV i.m., 3: WIV+CAF01 i.m., 4: WIV i.n., 5: WIV+ CAF09 i.n., 6: WIV+ CTA1-DD i.n., 7: WIV+ CTA1-3Me-DD i.n.
