# Supplementary material for: Cross-Protective Potential and Protection-Relevant Immune Mechanisms of Whole Inactivated Influenza Virus Vaccines Are Determined by Adjuvants and Route of Immunization
Source: Front Immunol. 2019 Mar 29;10:646. doi: 10.3389/fimmu.2019.00646 (PMC6450434; doi:10.3389/fimmu.2019.00646)
Supplement: Supplementary file 5 [file Presentation_5.PPTX]

## Slide 1
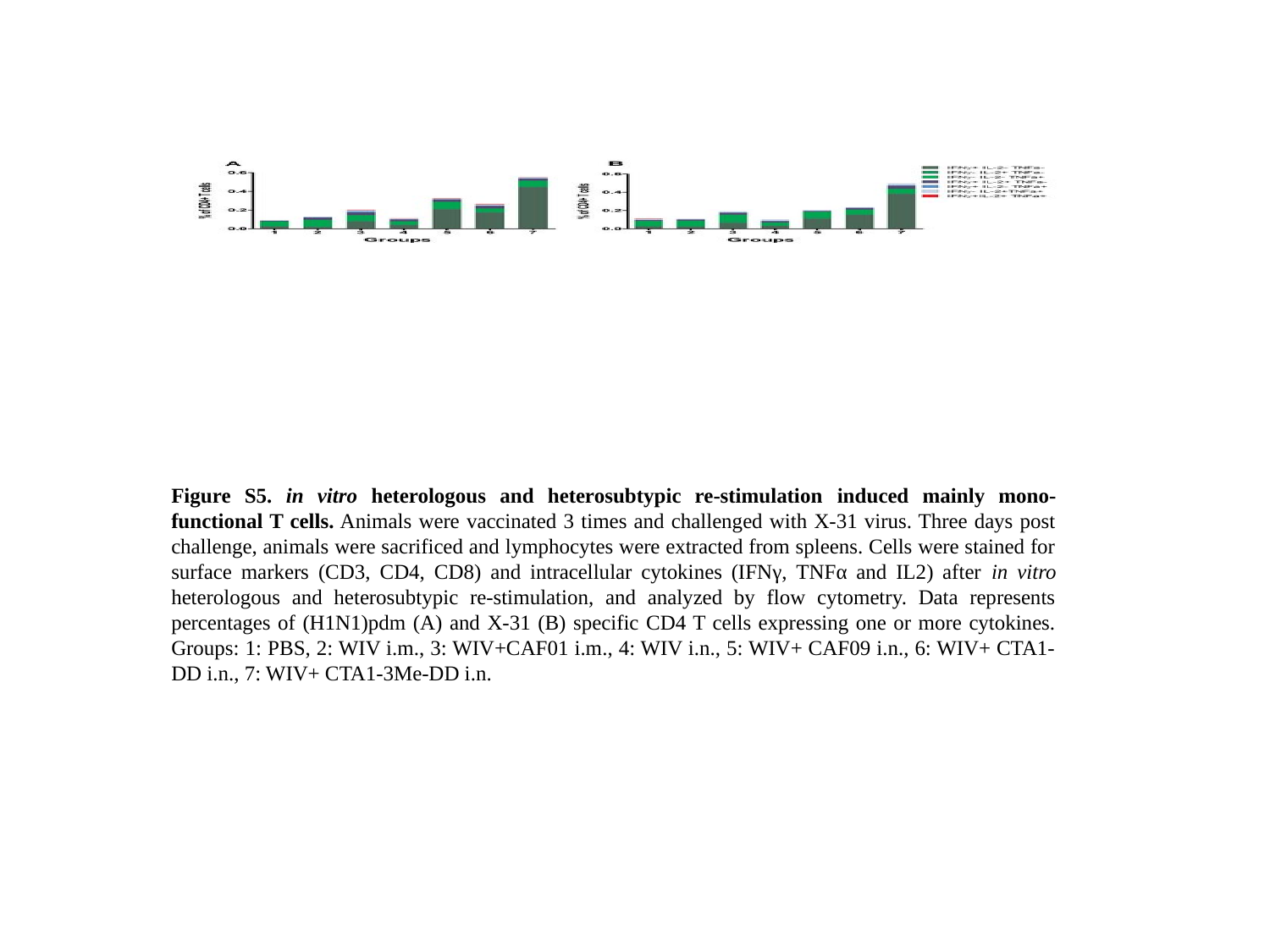

Figure S5. in vitro heterologous and heterosubtypic re-stimulation induced mainly mono-functional T cells. Animals were vaccinated 3 times and challenged with X-31 virus. Three days post challenge, animals were sacrificed and lymphocytes were extracted from spleens. Cells were stained for surface markers (CD3, CD4, CD8) and intracellular cytokines (IFNγ, TNFα and IL2) after in vitro heterologous and heterosubtypic re-stimulation, and analyzed by flow cytometry. Data represents percentages of (H1N1)pdm (A) and X-31 (B) specific CD4 T cells expressing one or more cytokines. Groups: 1: PBS, 2: WIV i.m., 3: WIV+CAF01 i.m., 4: WIV i.n., 5: WIV+ CAF09 i.n., 6: WIV+ CTA1-DD i.n., 7: WIV+ CTA1-3Me-DD i.n.
